# Comparison of Postural Stability and Balance Between Musicians and Non-musicians

**DOI:** 10.3389/fpsyg.2020.01253

**Published:** 2020-06-23

**Authors:** Manfred Nusseck, Claudia Spahn

**Affiliations:** Freiburg Institute for Musicians’ Medicine, Medical Center University of Freiburg, University of Music Freiburg, Faculty of Medicine at the Albert-Ludwigs-University of Freiburg, Freiburg Centre for Research and Teaching in Music, Freiburg, Germany

**Keywords:** postural stability, postural balance, posturography, pressure plate, instrumental postures

## Abstract

Good postural stability and balance provide a basis for optimal movement. For instrumental musicians, particular postures are demanded during long periods of playing and practicing. These postures can potentially also affect the postural control system even in situations when the musician is not playing the instrument. The goal of this exploratory study was to measure the postural stability and balance of instrument musicians in non-instrumental situations. By comparing these measures with a control group of non-musicians, postural differences were identified which can be ascribed to certain playing positions arising from playing the instrument. The measurement technique used was a pressure platform (Zebris force plate) to record static posturography. The postural sway of the center of pressure (COP) and the postural balance (body weight distribution) in a standing position were measured in 390 students including music students (*n* = 346) and a control group (*n* = 44). The analyses revealed significant differences for specific instrumental groups. Around 23% of the pianists, 25% of the upper strings players, and 33% of the guitar players showed a weight distribution significantly shifted more to the left compared to the control group (9%). In contrast, 23% of the lower strings players and 33% of the percussionists were found to stand more to the right side than the control group (5%). The results indicate that there are certain unbalanced postural patterns in musicians, outside of the music performance situation, which can be provoked by instrumental playing postures. As postural misalignments can lead to severe postural disorders in older age, preventive activities to improve postural stability and balance should be considered in instrumental education, not only during, but also outside of instrumental playing situations.

## Introduction

The physical and psychological demands of playing a musical instrument at a professional level bring about huge challenges for the musculoskeletal system. The complex and often repetitive tasks in musical performance are known to increase the risk of injury and incidence of health problems (Steinmetz et al., 2015; Kenny and Ackermann, 2016; Kok et al., 2016; Perkins et al., 2017; Wijsman and Ackermann, 2019). One particular aspect which causes health problems can be postural misalignment. Posture is generally defined as the orientation of the body in specific positions (Rosário, 2014). It can be described in stillness or during movement. Postural stability is the ability to control the body position in space for the purpose of movement and balance (Woollacott and Shumway-Cook, 2002). It is necessary for maintaining a static position and for assisting body coordination in dynamic position changes. Prolonged poor or incorrect postures can cause musculoskeletal disorders.

The playing of musical instruments requires controlled and adequate movements, which are often carried out in asymmetric and non-optimal playing positions. Prolonged movements and monotonous muscle work in the upper extremities and neck-shoulder regions, alongside uncommon postures, increase the risk of developing musculoskeletal problems among musicians (for a review, see Blanco-Pineiro et al., 2017). Among music teachers, a risk factor for postural misalignments was seen to be caused by asymmetric playing positions (Wahlström Edling and Fjellman-Wiklund, 2009).

By performing a medical examination, Ramella et al. (2014) found that 66.2% of 98 conservatory students displayed incorrect posture and beginnings of musculoskeletal pathologies in standing position without their instrument. When carrying the instrument, the amount of non-optimal postures increased up to 73.4%. Blanco-Pineiro et al. (2015) showed that the overall posture of 100 performing music students was defective in 70%. Steinmetz et al. (2010) found a relationship between insufficiencies in postural stabilization and manifestations of musculoskeletal pain in 93% of 84 musicians who attended a specialized clinic. In another study, by performing medical examinations with a focus on the pelvis in 42 professional pianists, Türk-Espitalier (2008) found 59% with a pelvic obliquity. The author found more frequent iliac crests on the right side compared to the control group (36%) and assumed that this had been caused by the intense pedal work of the right foot.

In a study of van Eijsden-Besseling et al. (1993) on 73 first-year conservatory students, 22% showed postural disorders in a clinical examination without their musical instrument. Although this prevalence was compared to a control group of medical students not significantly different, the authors found that postural misalignments increased up to 32% when playing the instrument.

Ramella et al. (2014) found that the years of practice and performing was significantly associated with incorrect posture and had a negative impact on the postural quality of musicians. Moreover, unphysiological postures can affect the quality of the musical performance (Blanco-Pineiro et al., 2017). Therefore, the correction of postural disorders should become an important part of the prevention of musculoskeletal pathologies among musicians.

Beside postural stability, another important aspect of human movement is postural balance. It is necessary to sustain the center of body mass within limits that prevent falling over. Different pathologies can arise from poor and unbalanced postures (Panjan and Sarabon, 2010). A fully symmetrical standing posture provides maximum stability. Hamaoui et al. (2011) found that increased muscular tension, which restricts postural chain mobility along the torso, is likely to disturb postural equilibrium. Therefore, frequently performing particularly off-balanced instrumental postures can cause muscular fatigue and may lead to a general postural imbalance.

Postural problems, however, can extend beyond playing. Performing on an instrument requires a specific playing position, which the player must somehow manage within the scope of consciously using an economic playing style. Good postural stability and balance is the base of support, which will minimize stress and maximize efficiency. Specific training, for instance, through body-oriented methods, can help to align well-adjusted and healthy playing postures (Spahn, 2017). However, it is also important to be able to re-align to a balanced posture when not playing or holding the instrument after a long duration of playing and practicing (Blanco-Pineiro et al., 2015). For instance, a good activity is performing particular exercises to properly cool down after playing. Without such activities, the body might grow accustomed to certain non-optimal postures, which endure even in situations outside of playing the instrument.

Therefore, the hypothesis of this study was that specific instrumental playing positions affect the player’s general posture even without the instrument. Prolonged playing and practicing in an instrument-related posture might cause considerable changes in the general posture, which can be measured through postural stability and balance.

The aim of this exploratory study was to investigate the characteristics of postural stability and balance in musicians outside of the instrumental playing situation using computerized platform posturography (see Monsell et al., 1997). Postural stability was determined by the amount of postural sway and postural balance by calculating the pressure distribution underneath both feet. The measures were then compared with a control group of non-musicians to evaluate specific instrument-related differences.

## Materials and Methods

### Posturography

Posturography is a technique that measures postural control and has proved valuable for indicating postural stability and balance (Ruhe et al., 2010). It has often been used for clinical intervention studies, especially as a measurement, which might be used to predict falls among older people (for a review see Piirtola and Era, 2006; Low et al., 2017).

In this study, the Zebris FDM-S force platform (Zebris Medical, Isny, Germany) was used. The participants stood on the force plate and the pressure distribution beneath the feet was recorded. As the pressure shifted, the postural sway path of the center of pressure (COP) was calculated. COP is a virtual measurement point describing the center of the pressure sensors in order to characterize the spatial distribution of pressure over time (Buldt et al., 2018).

COP provides values of the path length, the mean velocity, and the area of postural sway. These COP measurements appear to be equally reliable for investigating general postural stability and balance (Ruhe et al., 2010). Therefore, in this study, the area of postural sway was used as the dependent variable. This value was calculated with an ellipse of confidence (COP-EoC) drawn around the area of sway which included 95% of the COP path as given by the Zebris WinPDMs Software. A high value denotes a large area of postural sway, which indicates poor control of postural stability (Winter, 1995). In contrast, a low COP-EoC implies a rather tight and static posture with reduced swaying behavior. This could possibly be due to increased muscular tension causing postural immobility (Hamaoui et al., 2011). Considering the age, the COP-EoC was rather similar for people between 21 and 69 years and showed a mean value of 70 mm² (*SD* = 44 mm^2^) within this age (Pomarino et al., 2013).

It has already been shown that body weight is a predictor of postural stability. Hue et al. (2007) found with a regression analysis on the COP velocity that in 59 male participants with an age range from 24 to 61 years and a body weight range between 59 and 209 kg, body weight was the strongest predictor of postural stability. Thus, body weight seems to be correlated with postural instability. To test if this variable also predicts the area of sway in the current study population, a similar regression analysis on the COP-EoC value and body weight was conducted. Furthermore, other demographic variables, such as gender and age, were also included. Since Hamaoui et al. (2011) found that muscular tension can affect postural stability, a self-assessment question about feeling tense was also included (see below).

The results of Hamaoui et al. (2011) led to the assumption that doing extensive sports could have an effect on postural stability in such a way that performing more sports regularly can possibly lead to higher postural tension and with it to a smaller COP-EoC. Therefore, to investigate this variable for the current study sample, the amount of sports exercises that the participants provided (see below) were inserted to the regression analysis.

In addition to the regression analysis with the whole sample, a second regression analysis has been performed only for the musicians including additional music-related variables. One could expect that especially the amount of practicing and playing the instrument may provoke a postural bias. Consequently, the years of playing the instrument, the time practicing per day, and the average musical performances in a year have been included to the analysis.

One theory about postural balance depicts posture as being a swinging inverted pendulum. As such, it could be possible that a wider distance between the feet can result in a less swaying posture due to the larger standing area. Therefore, the distance and angle between both feet were also analyzed in order to predict COP-EoC. Both variables were measured visually from the images of the feet captured on screen. The distance between the centers of the feet had been provided by the software. The measured angle was the two-sided opening angle between the feet. There was a small but significant correlation between distance and angle (*r* = −0.19, *p* < 0.001) with smaller angles measured when the feet were farther apart.

Another outcome variable provided by the force plate was body weight balance, which is the percentage of limb load calculated by the pressure difference underneath both feet. This value indicates a possible imbalance in the postural equilibrium between the left and right sides. Such limb load asymmetry cannot be used to diagnose specific postural problems, but can imply a disturbance of the self-perception of standing in a balanced posture. This disturbance may be caused by frequently performing in non-optimal postures (Ramella et al., 2014).

In this study the postural balance value was categorized into balanced and imbalanced postures. Since humans permanently shift from side to side and are not designed to stand perfectly 50/50 on both feet, there is a range of percentages which can be defined as normal boundaries for postural balance. However, it is not clear to which extent these limits are considered to be out of balance. In healthy people, a range of 6% weight difference between left and right side has been found as a normal range (Badke and Duncan, 1983). But, other studies defined 5% overweight to one side (i.e., 55%) as a complete dominance of that particular limb (Hesse et al., 1996; Anker et al., 2008). Therefore, in this study, a weight difference of 8% (i.e., 54/46%) was considered to be in the normal range and more than 4% to one side was designated as a postural imbalance.

### Procedure

For the measuring procedure, the participants had to stand on the force platform without shoes for 20s. Participants were told to adopt a neutral and relaxed position without any restrictions on how to stand. This procedure was equal to Pomarino et al. (2013). Only extreme postures showing too large or too small a distance or angle between the feet were corrected to achieve standing postures where the feet distance equals more the width of the pelvis. The participants were also instructed to breathe normally during the measurement process. During all this time, they were not holding an instrument. COP and postural balance measures were averaged across the recording time.

Participants were measured in two different conditions. Most of the participants stood continuously on the platform across conditions, but in some cases, participants left the platform between the different conditions. In the first condition, participants had to stand in a natural position with the arms alongside the body. The standard body posture of the participants was recorded in this condition.

In the second condition, participants were asked to stretch out the arms to the front in a 90-degree angle to the torso. They had to hold their arms in this position for the complete duration of recording. This posture activates specific muscle areas such as the shoulder muscles (infra- and supraspinatus) and postural musculature. A sustained elevated arm position can lead to repressions of local muscle activity in the torso, the back and the belly and can result in higher muscular tension with regards to posture (Palmerud et al., 2000). It was expected therefore, that the COP-EoC would reduce in this condition compared to the arms by side condition.

For instrumental musicians, the arms are always required in instrumental playing, either to hold the instrument or to maintain sound production, or even for both uses. As some instruments cannot be held whilst standing on the force platform, the second condition was used to simulate a muscular activity in the arms that was standardized for all participants. The difference to the arms by side condition provides evidence of the effect of muscular tension on postural stability.

To facilitate the measurement of the music students, the force platform was installed in the entrance hall of the University of Music, Freiburg, and students were asked to voluntarily participate in the study. Once they had agreed, they were measured with the force platform and were asked to complete a questionnaire. The length of the total procedure was about 3–4 min. The control group was measured in the entrance area in front of a medical lecture hall in the Medical Centre, University of Freiburg, with an identical procedure as for the music group. All data collection was completely anonymous.

### Questionnaire

The questionnaire consisted of general questions on gender, age, body weight, and body height. Furthermore, participants were also asked if they had a problem with their musculoskeletal system that restricted them in their movement and activity range, which might potentially impair their body posture. If so, they were also asked to briefly describe the kind of problem they had.

A question about handedness was included for the musicians in order to determine their rate of left-handedness. Limb dominance was not included since no relationship has been found between limb dominance and preference of the side of weight (Hesse et al., 1996).

The participants were asked to rate their current feeling on a five-point scale [(1) physically relaxed, (3) as usual, and (5) physically tense. (2) and (4) had no predefined answers]. On another five-point scale, they had to indicate how often they participated in endurance sports [(1) several times in a week, (2) once in a week, (3) once every 2 weeks, (4) once in a month, and (5) never].

The musicians were asked to name their main instrument and to declare the years of how long they already played the instrument. Additionally, they had to estimate their amount of usual practice time (hours per day) and the number of concerts they normally performed per year. They were also asked about the degree course they were studying (Bachelor, Masters, or school teaching degree).

### Participants

The music group contained 346 music students at the University of Music, Freiburg. The average age was 23.4 years (*SD* = 4.5 years). 52.6% were female and 47.4% were male students (Table 1). They were studying either Bachelor (46.2%), Masters (30.0%) of Music, or with a pedagogical focus in teaching music at school (23.8%). The students spent on average 2.9 h (*SD* = 1.6 h) a day practicing and performed about 27.1 (*SD* = 41.9) concerts in a year.

**Table 1:** Description of the study sample (in brackets: standard deviation of the mean).

		Music group (*n* = 346)	Control group (*n* = 44)
Gender (% female)		52.6%	61.4%
Age in years		23.4 (4.5)	23.1 (2.9)
Body weight in kg	Female Male	58.8 (8.3) 71.8 (9.4)	60.0 (7.3) 78.6 (7.6)
Body height in cm	Female Male	168 (7) 179 (6)	170 (7) 183 (7)
Problem in the musculoskeletal system		32.9%	36.4%
Current feeling (1: relaxed - 5: tense)		2.7 (0.9)	2.3 (1.1)
Practice time in h/day		2.9 (1.6)	-
Average number of concerts in a year		27.1 (41.9)	-

The control group needed to be comprised of students similar in age to the musicians, but without any experience of playing an instrument. For that, 44 medicine students were recruited. Since there were medicine students who had learned and were currently played an instrument, they were asked before the measurement procedure whether they did play an instrument, and if affirmative, they were not included in the study. There were 61.4% female and 38.6% male medicine students with an average age of 23.1 years (*SD* = 2.9 years). The gender distribution and the mean age did not differ significantly between the music and the control groups. The average body weight and body height across genders differed significantly between groups [weight: *F*(1,361) = 7.337, *p* = 0.007, and *d* = 0.44; height: *F*(1,361) = 11.539, *p* = 0.001, and *d* = 0.55], with lower values in the music group.

A problem with the musculoskeletal system was reported by a third (33.3%) of all students. This amount did not differ significantly between the music and the control group. The problems described were similar in both groups and included either previous incidences of conditions such as tendovaginitis, accidents involving broken bones or joints, and torn ligaments, or permanent chronic symptoms, such as pain in the arm or knee joints, dorsal pain, slipped disc, or scoliosis.

In terms of their current emotional state, the students reported a mean value of 2.7 (*SD* = 1.0), which is slightly lower than the middle value of 3. Both groups differ significantly from each other with lower values in the control group indicating less self-perceived tension, *F*(1,348) = 8.837, *p* = 0.003, and *d* = 0.48. A value less than 3 was reported by 34.3% of all students and by 52.3% in the control group as well as 31.7% in the music group. No significant difference was found between the genders.

In total, 9.4% of the music students were left-handed, which did not differ from the general assumption of 10% left-handedness in the general population.

There was a significant difference in sports participation between the music and the control group, *χ^2^* = 28.387, *p* < 0.001, and *d* = 0.59. Where 72.7% of the control group reported participating in sports several times a week, only 35% of the musicians reported this frequency. Furthermore, 20.4% of the music group did not participate in sports at all, whereas all members of the control group took part in at least some sport. There was no significant difference between genders.

The musical instruments were categorized into several instrumental groups, which were then used for the analysis (Table 2). The piano group contained the cembalo (*n* = 4). The upper strings group consisted of violin (*n* = 54) and viola (*n* = 10). The lower strings group consisted of cello (*n* = 24) and double bass (*n* = 10). The woodwind group contained clarinet (*n* = 7), saxophone (*n* = 4), and oboe (*n* = 12). The brass group consisted of trumpet (*n* = 15), trombone (*n* = 8), and horn (*n* = 7). The flute group was kept separate from the woodwind group and includes low-pressure flutes such as the recorder (*n* = 10) and transverse flute (*n* = 14). The bassoon was also considered individually due to the difference in the instrument’s weight in comparison to the other woodwind instruments. The “others” group included harp (*n* = 6) and accordion (*n* = 3).

**Table 2:** Description of the musical instruments in the music group, number of musicians who played the instrument, percentage in the sample of musicians, and the mean years of playing the instrument with standard deviation (*SD*).

Instrumental group	*N*	Percent	Years (*SD*)
Piano	69	19.9%	15.5 (6.6)
Voice	39	11.3%	9.6 (3.4)
Upper strings	59	17.1%	15.9 (2.9)
Lower strings	34	9.8%	13.5 (4.5)
Woodwind	23	6.6%	13.1 (5.2)
Brass	30	8.7%	14.4 (2.1)
Guitar	18	5.2%	14.4 (4.6)
Percussion	9	2.6%	12.6 (3.4)
Flute	24	6.9%	13.6 (2.4)
Organ	17	4.9%	9.7 (3.5)
Bassoon	10	2.9%	11.9 (3.7)
Others	14	4.1%	13.3 (3.5)
Total	346	100%	13.7 (4.8)

On average, the musicians played their main instrument for more than 13 years (Table 2). No difference between the genders was found. There were significant differences in the mean years of playing the instrument across the instrumental groups, *F*(11,296) = 6.349, *p* < 0.001, and *d* = 1.27. The lowest mean years of practice were found for the vocal and the organ groups and the highest for the piano and the upper strings group.

### Data Analyses

SPSS (Version 26, Armonk, NY: IBM Corp.) was used for the data analysis. Descriptive statistics were calculated for each variable. Metrical variables were reported with mean and standard deviation of the mean (*SD*). Parametric comparisons of postural variables with nominal variables were performed using ANOVAs. When analyzing more than one variable, a MANOVA was used to avoid the likelihood of Type I errors. Where significant differences were seen, *post-hoc* analyses with Tukey’s HSD correction were undertaken. Contingency tables were used to assess the distribution differences of any non-parametric variables, and Chi-square statistics were reported. Two linear regression analyses with stepwise regression were performed separately on the COP-EoC for both conditions on the whole sample and only for the music group. The model statistics with explained variance (corrected *R* ^2^) and the beta coefficient with *t*- and *p*-values of the significant predictors were provided. The Wilcoxon signed-rank test was used to compare the changes of preferred standing side between conditions. Effect sizes were reported by using Cohen’s *d* (Cohen, 1988). The level of statistical significance was set to 0.05.

## Results

### General Analysis of the Posturography Measures

Across all participants, the mean COP-EoC was 38.9 mm^2^ (*SD* = 20.7 mm^2^), which was in the lower range of the mean value found by Pomarino et al. (2013) and within the boundaries of the standard deviation. The mean values for the music and the control group are shown in Table 3. In the arms by the side condition, the difference between the groups was not significant, [*F*(1,388) = 3.055, *p* = 0.081, and *d* = 0.28]. As expected, the COP-EoC reduced significantly in the arms outstretched forwards condition, *t*(389) = 14.427, *p* < 0.001, and *d* = 0.64. In the second condition, there was no significant difference in the COP-EoC between both groups, *F*(1,388) < 1.0. Furthermore, no significant differences were found between genders, for handedness and for having a problem in the musculoskeletal system in both conditions.

**Table 3:** Mean values of the centre of pressure ellipse of confidence (COP-EoC), the distance and angle between the feet for each group (in brackets: standard deviation of the mean).

	Music group (*n* = 346)		Control group (*n* = 44)	
	Arms by side	Arms outstretched	Arms by side	Arms outstretched
COP-EoC in mm^2^	38.3 (20.1)	26.9 (16.4)	44.1 (25.1)	25.6 (13.9)
Distance between the feet in cm	22.2 (4.7)	22.3 (4.7)	17.9 (3.0)	17.9 (3.2)
Angle between both feet	21.3° (11.3°)	22.0° (11.5°)	23.7° (8.6°)	24.9° (7.9°)

A multivariate ANOVA has been performed on the distance and the angle between both feet with the factors gender and group. The distance between the feet was across all participants 21.1 cm (*SD* = 4.5 cm). There was a significant difference between the music students and control groups, *F*(1,366) = 35.461, *p* < 0.001, and *d* = 0.96, with smaller distances in the control group. No significant effects were found for gender.

The participants stood on the platform with an average angle of 21.6° (*SD* = 11°) between both feet. Between groups, the angle difference was not significant, *F*(1,366) = 3.323, *p* = 0.069, and *d* = 0.29. However, there was a significant difference between genders, *F*(1,366) = 21.892, *p* < 0.001, and *d* = 0.49, with lower angles for the female participants (*M* = 18.0°, *SD* = 9.9°) compared to the male participants (*M* = 25.7°, *SD* = 10.1°).

To test for possible predictors of the COP-EoC, a linear regression analysis was performed with gender, age, body weight, the distance, and the angle between both feet, having a problem in the musculoskeletal system, the ratings of current emotional state, and the amount of sports exercises as variables separately for both conditions. As body height and body weight correlated with *r* = 0.78, height was not included in the regression analysis due to collinearity problems.

The regression model for the arms by side condition was significant, *F*(2,315) = 15.152, and *p* < 0.001, with an explained variance of 8.2%. Only two variables showed significant influence in the model: the angle between both feet (Beta = −0.427, *t* = −4.309, and *p* < 0.001) and the distance between the feet (Beta = −0.095, *t* = −4.251, and *p* < 0.001). Similarly, for the second condition with arms outstretched, the regression model was also significant, *F*(2,315) = 8.710, and *p* < 0.001, with an explained variance of 4.6%. The same variables as in the first condition were similarly found to have significant predictability (angle: Beta = −0.237, *t* = −3.216, and *p* = 0.001; distance: Beta = −0.056, *t* = −3.351, and *p* = 0.001).

A second linear regression analysis was conducted with both COP-EoC variables on the music group only. The included factors were the same as in the first regression analysis with the addition of the years of playing the instrument, the average practice time, and the average number of performances in a year. In the arms by side condition, the model was significant with three factors, *F*(3,262) = 8.839, and *p* < 0.001. The significant factors were the angle (Beta = −0.384, *t* = −3.821, and *p* < 0.001), the distance between the feet (Beta = −0.074, *t* = −3.212, and *p* = 0.001) and the years of playing the instrument (Beta = 0.480, *t* = 2.154, and *p* = 0.032). The explained variance was 8.2%. For the COP-EoC in the arms outstretched condition, the model was significant with two factors, *F*(2,269) = 5.011, and *p* = 0.007, and an explained variance of 2.9%. The two significant variables were the angle (Beta = −0.205, *t* = −2.564, and *p* = 0.011) and the distance between both feet (Beta = −0.045, *t* = −2.390, and *p* = 0018).

### Comparisons Between Instruments

**Figure 1:**
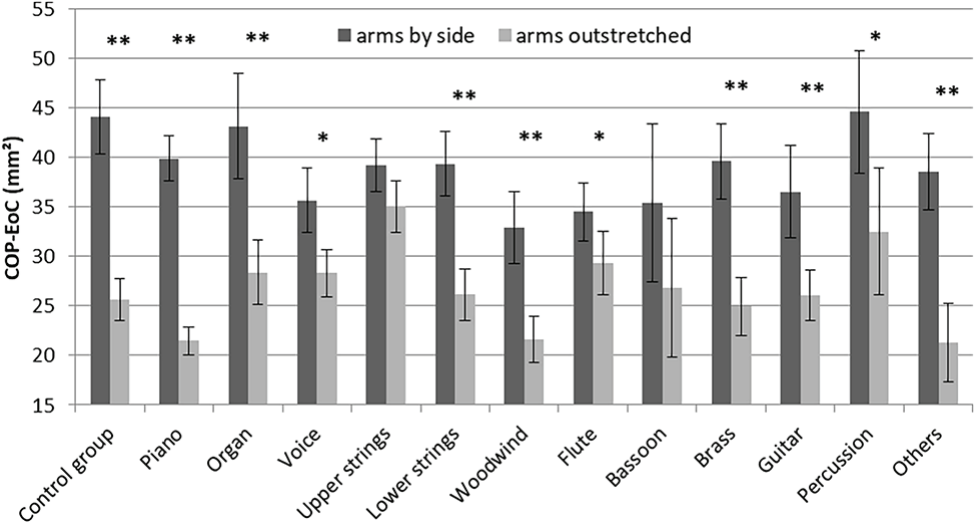
Mean values of the COP-EoC across instrumental groups (error bars present the standard error of the mean; the asterisks represent the statistics between the two conditions for each instrumental group; ^*^ *p* < 0.05; ^**^ *p* < 0.01).

The COP-EoC was compared using a multivariate analysis across the instrumental groups (including the control group) for both conditions. The mean COP-EoCs for each instrument are shown in Figure 1. In the first condition with arms by the side, no significant effect between the instruments was found, *F*(12,377) < 1.0. The second condition with arms outstretched showed a significant effect of instrument, *F*(12,377) = 2.606, *p* = 0.002, and *d* = 0.86. The *post-hoc* analysis yielded significant differences between piano and upper strings (*p* < 0.001) and woodwind and upper strings (*p* = 0.033) with respectively higher mean COP-EoC in the upper strings group.

As has been shown above, the COP-EoC reduced significantly between the two conditions. However, by separating the analyses into the different instrumental groups there were some instruments where COP-EoC did not decrease significantly, such as in the upper strings and the bassoon. Taking only the differences in COP-EoCs between the first and the second condition into account and comparing the difference value of each instrument with the control group, only the upper strings showed a significant difference (*post-hoc*; *p* = 0.001).

**Figure 2:**
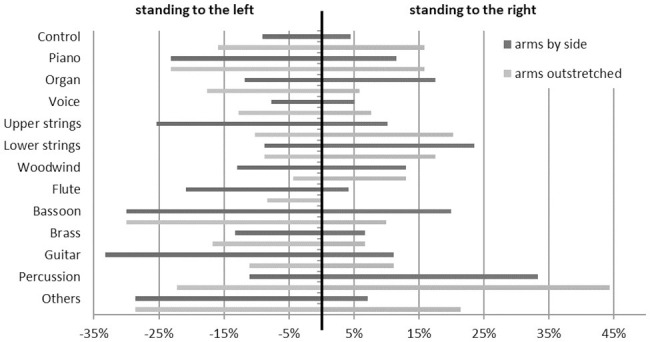
Percentages of the postural balance with standing more on the left or on the right foot divided by instrumental group and condition.

In the postural balance measurement, the mean percentage on the left foot across all participants was 50.3% (*SD* = 3.9%). There was no significant difference between the music and the control groups, genders, or instrumental groups. The balance values were split into a group of those standing more than 4% to the left side and a group of those standing more than 4% to the right side in order to achieve a more detailed analysis. The standard deviation of the mean percentage on the left foot across all participants of 3.9% confirms this previously defined classification of the borders within the range of a single standard deviation. The mean percentage of left/right postural balance for each instrumental group is shown in Figure 2. The center line represents equal weight distribution between the left and right feet.

The percentages of preferred sides were compared individually between each instrument and the control group. In the first condition, the piano group contained 23.2% participants standing to the left side. This was significantly different to the control group with 9.1%, *χ^2^*(113) = 6.161, *p* = 0.046, and *d* = 0.48. Similarly, in the upper strings group, there were more participants who stood to the left side (25.4%) than in the control group, *χ^2^*(103) = 6.318, *p* = 0.042, and *d* = 0.51. In the lower strings group significantly more people stood on the right foot (23.5%) compared to the control group with 4.5%, *χ^2^*(78) = 6.252, *p* = 0.044, and *d* = 0.59. A third of the guitar players (33.3%) stood on the left foot, which was significantly more than in the control group, *χ^2^*(62) = 7.074, *p* = 0.029, and *d* = 0.72. Moreover, a third of the percussionists (33.3%) stood significantly more on the right foot, *χ^2^*(53) = 7.470, *p* = 0.024, and *d* = 0.81. The bassoon players were significantly more off-balance (left: 20%; right: 30%) than the control group, *χ^2^*(54) = 6.728, *p* = 0.035, and *d* = 0.75. No significant distribution differences were found for voice, woodwind, brass, flute, and organ.

In the second condition with arms outstretched, no significant distribution difference of standing side preference was found between the instrumental groups and the control group.

Between the conditions, there was no significant difference in the percentage distributions across all participants. The control group seemed to stand more off-balance in the second condition compared to the first condition but without significance (Wilcoxon; *z* = 1.711, *p* = 0.087, and *d* = 0.53). The upper strings group shifted the postural balance in the second condition significantly to a more balanced position than in the first condition (*z* = 2.045, *p* = 0.041, and *d* = 0.55). Similarly, more balance changes but with without significance have been found for the guitar (*z* = 1.947, *p* = 0.052, and *d* = 1.03) and the flute groups (*z* = 1.890, *p* = 0.059, and *d* = 0.84).

## Discussion

This study investigated parameters of postural stability and balance in musicians outside instrumental playing situations, and compared these with a control group of non-musicians. Both the music and control groups were very similar in age and gender. The average prevalence of muscular problems was about one-third in both groups. For the musicians, this corroborates findings of other prevalence studies (see Wijsman and Ackermann, 2019). The music group contained participants of significantly less body height and weight. This could be due to the multicultural consistency of the music group. However, socio-cultural background information was not collected in this study.

The control group participated in considerably more sports activities than the music group. The control group consisted of medical students who may use endurance sports to compensate for long times spent sitting and studying. In contrast, participants in the music group were practicing their instrument on average for 3 h a day. It is therefore possible, that the practicing, which can be physically rather demanding, caused the reduced sports behavior. However, research investigating the reasons behind these different behaviors has not yet been undertaken.

### Postural Stability

Posturography was found to be a simple and effective technique for measuring postural stability and balance. Postural stability was defined by the swaying of the COP and was calculated with an ellipse of confidence drawn around the sway path (COP-EoC). All participants were found to lie within the normal range of postural stability indicated by Pomarino et al. (2013). Linear regression analysis of the COP-EoC to identify possible predictor variables showed no effect of body weight. This result was in contrast to Hue et al. (2007), a difference which could possibly be caused by the fact that this study did not contain people of extreme sizes. Furthermore, the self-perceived feeling of tension was also not a significant predictor of COP-EoC. This suggests that the perception of tension is not related to specific objective aspects of postural stability. Only the distance and the angle between both feet were found to be significant predictors of COP-EoC. The larger the angle and distance, the lower the COP-EoC. However, the explained variances in the regression models were very small, indicating that the effect of these variables was rather negligible. Moreover, there were no extreme distances and angles measured due to the space limitation of the force plate itself. More systematic research investigating the relation between the distance and the angle between feet on the amount of postural sway is needed.

The regression analysis only with the music group yielded a significant association of the years of practicing and playing on the COP-EoC in the arms by side condition. The longer the participants played their instrument, the larger was the COP-EoC value. Ramella et al. (2014) found a negative impact of the years of playing on postural disorders. In our study, there is no indication that the increase of the COP-EoC can be interpreted as a negative effect. It may be possible, that the participants with more years of playing were more relaxed compared to the participants with less years of playing. However, the range of the COP-EoC changes were rather small and the explained variance of the regression model is exiguous. Since the years of playing disappeared in the regression analysis of the second condition with outstretched arms, the effect of this variable seems to be negligible small. To investigate a possible impact of the years of playing, a larger sample with a greater extent of older musicians would be helpful.

In agreement with Hamaoui et al. (2011), the COP-EoC reduced considerably in the condition with outstretched arms. This finding indicates that muscular stress in the musculoskeletal system regarding the arms caused a more static and strained posture. Interestingly, this was not the case for the upper strings musicians. This could be due to the fact that they are used to muscular activities in the arms while playing in a standing position. Therefore, the postural stability was less influenced based on this expertise. The vocalists and the flute players also showed an indication of an absence reduction of COP-EoC in the second condition. However, there are other instruments such as the woodwind and the brass, which are also played often in upright position with certain arm activities that did reduce in COP-EoC. More research on the particular playing behaviors is needed to certify this finding.

### Postural Imbalances

In the investigations of postural imbalances in the standing position, a value of 4% more on one body side was used as the threshold value denoting a preference for one side. The percentage of participants preferring one body side were calculated in each instrumental group and compared with the control group.

**The pianists** showed a higher percentage of standing to the left side. This could be caused by the high demands on the right foot in playing the pedal, as suggested by Türk-Espitalier (2008). When operating the right pedal, the left body side and the left hip muscles experience more stress in order to maintain the playing position. This could lead to an imbalance in the general posture. This assumption is supported by findings amongst the organ players who did not differ in side preference to the control group. The organ is played in a similar manner to the piano, in a seated position, but with more activity in both legs. A balanced playing posture, therefore, requires certain back and hip muscle areas to ensure unrestricted movements in the arms and legs, which may possibly prevent an imbalance in the general posture.

A quarter of the **upper strings players** stood more to the left side. An obvious explanation for that might be the fact that the instrument is normally held on the left side. The left body side may, therefore, be adapted to perform a holding position and to allow the right arm to move freely along the bowing paths. Without the instrument, the left side might still remain an active part of maintaining the playing position.

In contrast to the upper strings, the **lower strings players** were seen to stand more often with a preference for the right foot. For these instruments, the right body side is also the bowing side while the instrument leans over the left shoulder. Therefore, the left side performs a similar role to that in the upper strings instruments. Since lower strings instruments stand on the ground, holding the instrument is a minor matter. However, due to the size of these instruments, the bowing movement in the right arm is larger and players often lean over the right side to provide optimal bowing and expressive movement. This might have stabilized the right body side more than the left side and possibly affected the postural imbalance to the right.

The **classical guitar** is played in a sitting position and rests on the left leg. The left side and the left leg are involved in holding the instrument. The right leg is often opened to the right side for stabilization. In standing position, it might be possible that the right leg pushes the body slightly to the left.

The interpretation of the finding that the **percussionists** preferred to stand on the right foot is more difficult. It was expected that there would be no difference, as percussionists often play several instruments such as the vibraphone, the timpani, the snare drum, and even the drum kit. All of these instruments apply different playing positions which use different body areas and movements. The most common activity between these instruments (except for the snare drum) is that the right foot is used to play a pedal. However, this would assume according to the findings above that the players might stand more to the left side. However, for the vibraphone, for instance, the pedal has to be played in standing position and the right leg has to perform both pedaling and standing activities simultaneously. This could possibly result in a more active right side in the general posture. At least, more information from each player about which of the percussion instruments they play most frequently are necessary in order to properly interpret the result.

Within the woodwind instruments, the **bassoon** is the largest and is played with the aid of a carrying strap around the neck. It lies diagonally across the player with the lower end to the right side. The right arm is, therefore, shifted slightly backward. The bassoon players in this study were seen to have more off-balanced postures than the control group. It is unclear here how the players held the instrument to play since it can be played in a sitting or standing position. More details about particular playing postures are needed to interpret this finding.

The other **woodwind instruments**, **the brass**, **the flute**, and **the voice** showed no difference in general posture compared to the control group. This could be due to the fact that these instruments are mainly played in a standing position. Also, these instruments are often referred to as symmetrical instruments (Wahlström Edling and Fjellman-Wiklund, 2009) despite the fact that there were some instruments included in these groups which can be described as more asymmetrical, such as the transverse flute and the trombone. Nevertheless, it seems that these players have no specific instrumental position that influenced the general posture.

All significant differences in the preference for standing side between the instrumental groups and the control group disappeared in the second condition with outstretched arms. This was mainly caused by the considerable changes in the control group between the conditions. The variance increased and no statistical test reached significance. However, the control group reduced the COP-EoC in the second condition but increased the variance in balance. The restricted mobility possibly amplified the preferred standing side in agreement with Hamaoui et al. (2011). In the control group, this could be on both sides.

For the musicians, an amplification of the preferred standing side would lead to the assumption that a greater number of participants would stand on their instrument-related preferred side in the second condition. However, this was not the case. In contrast, some instrumentalists even improved their postural balance compared to the first condition. This might be explained by the fact that musicians are used to practicing to perform using the most economic posture, i.e., a balanced position, and with the outstretched arms condition, the body reacted unconsciously, possibly by moving into a good playing position. To investigate this finding in more detail, movement and posture analysis should be performed using for instance 3D digital Motion Capture systems.

One might think that most of the main findings on differences in postural balance could be explained by considering the symmetrical and asymmetrical instrument holding postures. These postures have been found to be a certain predictor for postural pain (Wahlström Edling and Fjellman-Wiklund, 2009; Ramella et al., 2014). However, it is not trivial to determine which instruments can be clearly defined as symmetric or asymmetric. Although the violin is mainly considered to be an asymmetric instrument whilst playing the instrument, postural balance has been found to be very symmetric (Spahn et al., 2014). In contrast, the clarinet is specified as a symmetrical instrument, but when played in a sitting position, postural balance is considerably shifted to the right side (Arnold, 2015). Therefore, it remains unclear how to define the proper symmetrical component of any individual instrument. It is also necessary to take the musician’s usual playing and practicing position into account.

Another possibility would be to interpret the results regarding the fact that musicians perform either in sitting or standing position (Blanco-Pineiro et al., 2017). While some instruments certainly require a sitting position (such as piano, organ, guitar, cello, and tuba), most of the other instruments can be played in both seated and standing positions. For these instruments, it is up to the musician if the most preferred position is sitting or standing or even both equally. Therefore, to take this variable into account, more details on the particular playing position of the musicians are needed.

### Limitations of the Study

A particular limitation of this study was the sample sizes of the individual instrumental groups. Even though the study contains a large total sample size, the division into different instrumental groups resulted in some rather small group sizes. It would be necessary to enlarge these instrumental groups to adequately draw any conclusions about postural stability and balance for these instruments. However, it is quite difficult to find a reasonable number of music students playing particular instruments, meaning that a multicenter study at a number of different Universities of Music would be beneficial.

Additionally, many music students also play more than one instrument. At the University of Music, pianists and guitarists do not have to learn another instrument, where all the other instrumentalists have to study to play piano. There could be the possibility of an influence of the second instrument on the postural bias. However, for music students, it can be assumed that in most of the cases, it is negligible in comparison to the main instrument. To investigate effects of an additional second instrument causes complex analyses of similarities and differences between both instruments and the documentation of the practice time on both instruments would require a larger sample size.

Measuring the amount of postural sway and postural balance can only provide limited insights into the actual posture of the person or diagnosis of possible postural disorders. There are certain direct relations, such as the fact that neuromusculoskeletal disorders generally result in a degenerated balance system (Winter, 1995). However, not all postural imbalances are caused by postural disorders. In postural control, two kinds of origins can be distinguished, either anatomical or functional causes (Blaszczyk et al., 2000). In younger adults, some anatomical geneses are functionally compensated. In this study, clear imbalances were found in some instrumental groups, indicating evidence of functional reasons produced by holding the musical instrument. Musculoskeletal measurements should be included in further research in order to gain more details about the relation of instrument holding with actual postural misalignments.

## Conclusion

The posturography used in this study has been shown to be a useful measurement technique for investigating postural stability and balance in musicians. The results demonstrated certain postural differences in musicians compared with a control group, many of which can be explained by specific instrumental playing postures. Prolonged activities in one particular posture can affect the musculoskeletal system and consequently generate postural misalignments. An optimally controlled postural system provides an equal margin of maximum stability and balance (Blaszczyk et al., 2000). It has been shown that in older people, former postural problems become strengthened. In this study, even healthy music students already showed considerable postural deficits. Performing a similar study with professional orchestra musicians with more playing experience would be of further interest. Consequently, preventive activities to train toward stable and balanced posture, not only while playing, but also when not playing the instrument, should be considered early in the instrumental education process.

## Data Availability Statement

The datasets generated for this study are available on request to the corresponding author.

## Ethics Statement

Ethical review and approval was not required for the study on human participants in accordance with the local legislation and institutional requirements. Written informed consent for participation was not required for this study in accordance with the national legislation and the institutional requirements.

## Author Contributions

Both authors contributed extensively to the work presented in this paper. They contributed to the conception, design of the study, the data collection, and statistical analyses. The authors co-wrote the manuscript.

## Conflict of Interest

The authors declare that the research was conducted in the absence of any commercial or financial relationships that could be construed as a potential conflict of interest.
